# Effects of N-acetylcysteine on spexin immunoreactivity in kidney tissues of rats treated with adriamycin

**DOI:** 10.22038/IJBMS.2023.71942.15635

**Published:** 2024

**Authors:** Tuba Yalçin, Tuncay Kuloğlu, Nalan Kaya Tektemur, Ahmet Tektemur, İbrahim Enver Ozan

**Affiliations:** 1 Batman University, Health Services Vocational School, First and Emergency Program, Batman, Turkey; 2 Firat University, Medicine Faculty, Department of Histology and Embryology, Elazığ, Turkey; 3 Firat University, Medicine Faculty, Department of Medical Biology, Elazığ, Turkey

**Keywords:** Adriamycin, N-Acetylcysteine, Nephrotoxicity, Neuropeptide Q, Spexin

## Abstract

**Objective(s):**

Due to its negative side effects, mainly nephrotoxicity, adriamycin (ADR) is used fairly infrequently. The purpose of this study is to investigate the effects of N-acetyl cysteine (NAC) on the immunoreactivity of spexin (SPX) in the kidney tissues of rats given ADR.

**Materials and Methods:**

A total of 28 male Sprague-Dawley rats were randomly assigned to four groups (n=7): control (no intervention), NAC (150 mg/kg/day, administered intraperitoneally), ADR (single dose of 15 mg/kg, administered intraperitoneally), and ADR+NAC (single dose of 15 mg/kg ADR + 150 mg/kg/day NAC, both administered intraperitoneally). The experiment was concluded on the 15^th^ day.

**Results:**

The administration of ADR resulted in biochemical and histopathological alterations in the kidney. It was found that ADR treatment led to elevated levels of TOS (total oxidative stress), apoptosis, and SPX. Conversely, when NAC was administered as a treatment, it effectively reduced TOS, apoptosis, and SPX levels. These findings suggest that SPX may contribute to the development of ADR-induced kidney damage.

**Conclusion:**

Further investigations are warranted to gain a comprehensive understanding of kidney damage, and specifically to elucidate the role of SPX in this context. Additionally, these studies can pave the way for exploring novel therapeutic strategies targeting SPX to prevent and/or treat the development of kidney damage.

## Introduction

ADR, a potent drug belonging to the anthracycline group, exhibits notable cytotoxic effects against various solid tumors, including hematological malignancies, ovarian cancer, breast cancer, and testicular cancer (1). However, the therapeutic application of ADR is limited due to its known toxic side effects on various organs, including the kidney (2). Kidney injury induced by drugs is a significant concern associated with the use of chemotherapeutic agents. ADR-induced kidney injury is mediated through mechanisms such as oxidative stress and apoptosis (3, 4). Oxidative stress-induced kidney damage can be mitigated at the cellular level through the actions of anti-oxidant enzymes, including reduced glutathione (GSH), catalase (CAT), and superoxide dismutase (SOD) (5-7). SOD converts the superoxide radical (O_2_-) into hydrogen peroxide (H_2_O_2_), which is subsequently broken down into H_2_O and O_2_ by CAT, and further reduced to H_2_O by the enzyme glutathione peroxidase (GPx). These enzymatic reactions play a protective role against reactive oxygen species (ROS) (4, 8, 9). Decreased activity of these enzymes leads to a reduction in the overall anti-oxidant capacity (4, 8, 9). Studies have shown that disruption of the balance between total anti-oxidants and oxidants contributes to the development and progression of ADR-induced nephrotoxicity. Specifically, these studies have reported a significant increase in malondialdehyde (MDA) levels in the ADR group compared to the control group, along with significant decreases in CAT activity, total anti-oxidant status (TAS), and GSH levels (4, 6). Furthermore, it has been established that DNA damage is one of the adverse effects of ADR (10, 11). 

N-acetyl cysteine (NAC) is a thiol-containing amino acid that is recognized for its ability to neutralize free radicals, inhibit lipid peroxidation (LPO), and prevent the depletion of glutathione (GSH) (12, 13). Additionally, NAC possesses metal chelation activity, which enables it to regulate or rectify the imbalance between pro-oxidants and anti-oxidants (14). Furthermore, studies have demonstrated that NAC exerts protective effects against nephrotoxicity by reducing inflammation and oxidative stress (15,16). 

The recently discovered endogenous neuropeptide SPX, also referred to as neuropeptide Q, controls a number of physiological functions, including digestion, gastrointestinal motility, obesity, diabetes, energy metabolism, salt/water balance, and cardiovascular and renal functions (17). Numerous organs, including the skin, lung, stomach, small intestine, colon, liver, pancreatic islets, thyroid, adrenal gland, and kidney, have shown evidence of intracellular SPX immunoreactivity (18). Additionally, research has shown that SPX can reduce renal oxidative stress and inflammation in those with obesity-related renal impairment (19).

In light of this context, despite the frequent clinical use of adriamycin either alone or in combination with other antineoplastic agents, it has been established that it induces significant histological and biochemical alterations in rat kidney tissue. Therefore, there exists a necessity for an effective agent capable of mitigating or reversing this damage. The goal of the current study is to evaluate how NAC, a promising anti-oxidant with the ability to prevent apoptosis and free oxygen radical damage, affects kidney tissue. Furthermore, the study aims to investigate the effects of adriamycin on SPX immunoreactivity following kidney injury and subsequent treatment with NAC.

## Materials and Methods

***Animals***

The recently discovered endogenous neuropeptide SPX, also referred to as neuropeptide Q, controls a number of physiological functions, including digestion, gastrointestinal motility, obesity, diabetes, energy metabolism, salt/water balance, and cardiovascular and renal functions (17). Skin, lung, stomach, small intestine, colon, liver, pancreatic islets, thyroid, adrenal gland, and kidney are just a few of the tissues that have shown intracellular SPX immunoreactivity (18). Additionally, research has shown that SPX can reduce renal oxidative stress and inflammation in those with obesity-related renal impairment (19). The rats were fed pellet rat food, and water was available to them at all times. All experimental methods complied with relevant ethical standards for the use and treatment of laboratory animals. When employing animals in the study, the requirements for ARRIVE (Animal Research: Reporting of *In Vivo* Experiments) were adhered to.

***Drug preparation and experimental design***

The study’s rats were divided into four groups at random: control, NAC, ADR, and ADR+NAC (n=7 for each group). Throughout the 14-day study period, the control group received no interventions. ADR, purchased from Saba Farma, Turkey, was prepared by reconstituting lyophilized ADR powder with sterile water for injection to achieve a final concentration of 2 mg/ml. On the first day of the experiment, a single intraperitoneal (IP) dose of ADR was administered. In the NAC and ADR+NAC groups, liquid NAC (Husnu Arsan Farma, Turkey, supplied Asist® 300 mg/3 ml, 10% solution) was administered IP at a dose of 150 mg/kg every other day for 14 days. The dosage and duration of the drugs used in the study were based on relevant literature (20,21,22), following the recommendations of Altinkaynak *et al*. All groups of rats were euthanized under anesthesia at the conclusion of the experiment by administering IP doses of ketamine (75 mg/kg) and xylazine (10 mg/kg) (23,24,25). A 10% formaldehyde solution was used to quickly remove certain kidney tissues for histological analysis. Blood samples were collected and transferred to centrifuges operating at 4000 rpm for 5 min. The remaining kidney tissue samples and the resultant serum were kept at -20 °C until further examination.

***Tissue processing and histomorphological analysis***

For histopathological evaluations, the kidney tissues were fixed in 10% formaldehyde for 24 hr. Subsequently, the standard tissue processing protocol was followed (22). Five-millimeter pieces with a thickness of paraffin were created. Then, Masson trichrome and Hematoxylin-Eosin (H&E) stains were used to color the sections. They were examined using a light microscope (Leica DM500), and the results were captured on camera (Leica DFC295; Wetzlar, Germany). Histopathological changes were assessed and graded on a scale of absent (0), mild (1), moderate (2), and severe (3) based on their severity. A cumulative histopathological evaluation score was calculated accordingly.

***Immunohistochemical evaluation***

The avidin-biotin-peroxidase complex technique, as previously described (22,26), was used to assess SPX immunoreactivity in kidney tissue. Following customary histological practices, 5 m-thick sections were produced on polylysine-coated slides and exposed to antigen retrieval by microwave treatment in citrate buffer solution (pH 6.5) for 12 min. The endogenous peroxidase activity was inhibited with a hydrogen peroxide blocker (Hydrogen Peroxide Block, TA-125-HP, Lab Vision Corporation, USA). The sections were subsequently subjected to Ultra V Block (TA-125-UB, Lab Vision Corporation, USA) for 5 min to limit nonspecific binding. The sections were then incubated with SPX primary antibodies (A04088-1, Boster Biological Technology, Pleasanton, CA, USA) for 60 min at room temperature in a moist, dark environment. The secondary antibody (TP-060-BN, Thermo Scientific, England) was then incubated before the Streptavidin Peroxidase (HRP) enzyme (TS-060-HR, Thermo Scientific, England) was added. Mayer’s Hematoxylin was used as the counterstain. Following a PBS and distilled water wash, a mounting media was applied to the sections. The prepared slides were examined under a light microscope (DM 500, DFC295, Leica, Germany), evaluated, and photographed. The immunohistochemical assessment was performed based on the prevalence (0.1: <25%, 0.4: 26–50%, 0.6: 51–75%, 0.9: 76–100%) and intensity (0: none, +0.5: very little, +1: little, +2: moderate, +3: severe) of staining. A histoscore was calculated by multiplying the prevalence and intensity values (27).

***Terminal deoxynucleotidyl transferase dutp nick-end labeling (TUNEL) test method***

Apoptotic cells in ADR-damaged kidney tissues were identified using the TUNEL method (In Situ Apoptosis Detection Kit, Chemicon, cat no: S7101, USA). TUNEL staining allows for the detection of apoptotic cells by staining the nuclei. Normal nuclei stained green with methyl green, while apoptotic cells exhibited brown nuclei staining. In randomly selected areas of each group, a total of 500 cells were counted under x10 magnification. The number of cells that have undergone apoptosis was divided by the total number of cells (normal + apoptotic), yielding the apoptosis index (AI), which is expressed as a percentage (28).

***Biochemical analyses***

*Blood samples*

After the experiment, blood samples were collected, and the serum was extracted by centrifuging the blood for 5 min at 4000 rpm. The obtained serum samples were placed in 1.5 ml centrifuge tubes and stored at -20 °C in order to measure the activity of kidney enzymes.

*BUN and creatinine levels in serum*

Renal function and serum levels of renal enzyme activities were assessed in the study. A commercially available kit and an autoanalyzer (Advia 2400, Siemens, Germany) were used to measure the levels of blood urea nitrogen (BUN) and creatinine.

*Determination of SPX, TAS, and TOS levels*

After adding PBS (pH 7.4), the kidney tissue samples were homogenized using the Bullet Blender® Homogenizer apparatus. Following centrifugation at 2,000–3,000 rpm for 20 min at +4 °C, the supernatants were carefully collected. The supernatants were then divided for use in the ELISA assay. Using commercial enzyme-linked immunosorbent assay (ELISA) kits, serum, and kidney tissue samples were examined for SPX levels as well as total oxidant/anti-oxidant (TAS and TOS) levels in accordance with the manufacturer’s recommendations. The measurements were carried out using a 96-well plate reader (Multiskan FC, Thermo Scientific, USA) at a wavelength of 450 nm. Rat-specific ELISA kits were utilized, including SPX (Lot: 2022105, Shanghai Coon Biotech, Shanghai, China), TAS (AD3283Ra, AndyGene Rat Total Anti-oxidant Status (TAOS) ELISA Kit/301 University Village Drive Richardson, TX75081, USA), and TOS (AD3282Ra, AndyGene Rat Total Oxidant Status (TOS) ELISA Kit/301 University Village Drive Richardson, TX75081, USA). The test results were expressed in pg/ml, U/ml, and pg/ml, respectively, for SPX, TAS, and TOS. The SPX assay had a test range of 79 pg/ml to 5000 pg/ml with a sensitivity of 55.36 pg/ml. The TAS assay had a sensitivity of 0.1 U/ml and a test range of 0.2 to 8 U/ml. The sensitivity level of the TOS assay was 0.7 pg/ml, and the test range was 3.75–120 pg/ml.

***Statistical analysis***

Using software from the SPSS 22.0 package, the study’s data were statistically evaluated. The median values for the numerical data were shown alongside the minimum and maximum values. The Shapiro-Wilk test was employed to assess the normal distribution of variables. The Kruskal-Wallis test was used for the overall comparison of more than two groups. The *post-hoc* Dunn test was used to compare paired groups after the Kruskal-Wallis test. A 0.05 *P*-value was deemed statistically significant. The GraphPad Prism 9.3.1 application was used to create the graphs.

The sample size for each group was calculated using G Power Analysis using G*Power version 3.1 (University of Kiel, Germany) in this investigation. A sample size of 7 animals per group would be sufficient to obtain a type 1 error of 0.05 and a power of 0.95, according to the power analysis (29).

## Results

***Effects of NAC treatment on the histopathology of the rat kidney samples following ADR treatment***

**Figure 1:**
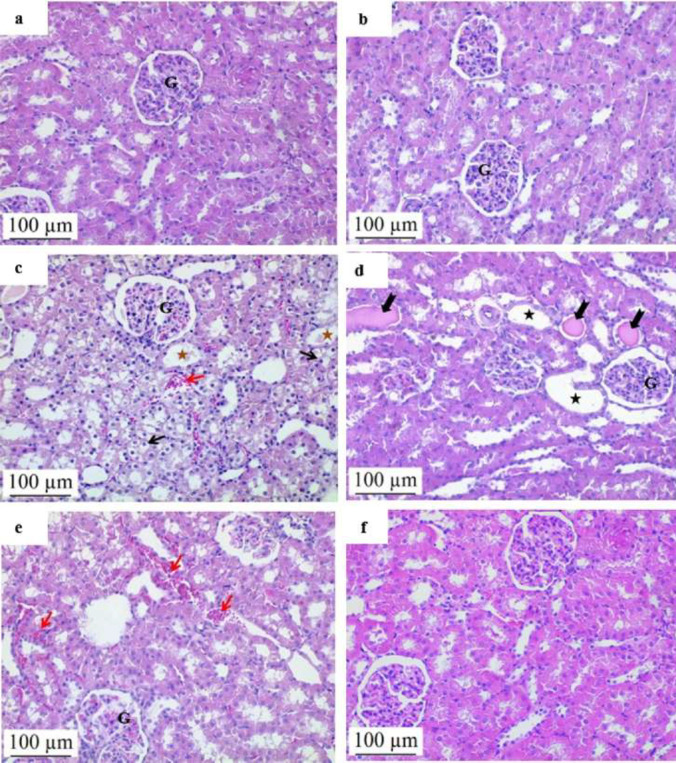
Effect of ADR and/or NAC applications on kidney tissue histopathology in rats

**Figure 2:**
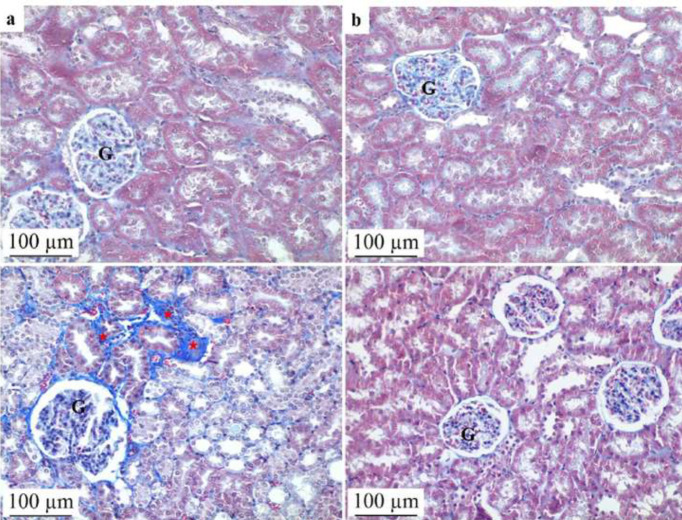
Effect of NAC treatment on kidney tissue histopathology after ADR application in rats

Based on microscopic examination, the kidney sections from the control and NAC groups showed no abnormal histopathological findings. On the other hand, the ADR group exhibited significant histopathological changes in the kidney tissue compared to the control group. These changes included edema, hyaline cast formation, tubular dilatation, fibrosis, tubular vacuolization, and erythrocyte extravasation. The ADR group significantly outperformed the control group in terms of total histopathological evaluation scores. The histological evaluation score in the ADR+NAC group, however, was considerably lower than in the ADR group, demonstrating that NAC therapy had a mitigating impact on ADR-induced kidney damage (Table 1). These results were supported by statistical analysis, which revealed a substantial distinction between the ADR and ADR+NAC groups (*P*=0.001) (Figures 1 and 2). 

***SPX immunoreactivities following exposure to ADR and subsequent NAC treatment in rat kidney tissue***

**Figure 3:**
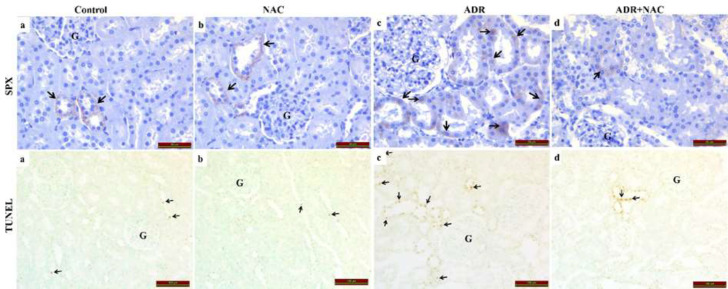
SPX immunoreactivity/TUNEL staining photomicrographs of renal tissue after injection of ADR and/or NAC

In Figure 3, the immunohistochemical staining for SPX immunoreactivity in kidney tissues is shown. There was no discernible change between the SPX immunoreactivity of the NAC group (a) and the control group (b) (*P*=0.75). The ADR group (c) showed a statistically significant increase in SPX immunoreactivity when compared to the control group (*P*=0.004), indicating up-regulation of SPX in response to ADR-induced kidney damage. However, the ADR+NAC group (d) had statistically significantly reduced SPX immunoreactivity compared with the ADR group (*P*=0.004).

***Effects of ADR and/or NAC application on the apoptotic index in kidneys***

In Figure 3, apoptotic cells in the kidney tissues were found using TUNEL labeling. It is evident that NAC therapy alone did not cause apoptotic cell death because there was no statistically significant difference between the TUNEL positive in the NAC group (a, b) and the control group (*P*=0.706). In contrast, the TUNEL positivity in the ADR group (c) was significantly higher than in the control group (*P*=0.001), demonstrating an increase in apoptotic cells in response to the kidney injury caused by ADR. The TUNEL positivity was significantly lower in the ADR+NAC group (d) compared to the ADR group (*P*=0.006), suggesting that NAC treatment decreased the number of apoptotic cells produced by ADR. These results suggest that NAC has a protective effect against apoptosis in the context of ADR-induced kidney injury.

***ADR and/or NAC administration effects on renal tissue biochemical parameters***

**Figure 4:**
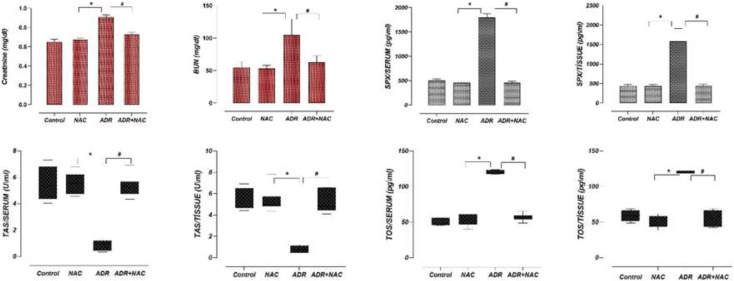
Effects of ADR and/or NAC treatment on creatinine, BUN, SPX, TOS and TAS levels in rats

In the biochemical investigation, serum levels of creatinine and BUN were analyzed in all groups. Between the NAC group and the control group, there were no statistically significant differences in the levels of creatinine or BUN (*, *P*=0.696, *P*=0.770, respectively) (Figure 4). The ADR group had significantly higher levels of creatinine and BUN than the control group (*P*=0.001 and *P*=0.001, respectively) (Figure 4), indicating that the ADR medication caused renal impairment. However, in the ADR+NAC group, there was a significant decrease in creatinine and BUN levels compared to the ADR group (#, *P*=0.027, *P*=0.023, respectively) (Figure 4, Table 2).

***ADR’s effects on kidney tissue’s oxidative stress measures and SPX levels***

Similar TAS results were found in serum and kidney tissue samples from the control and NAC groups in the analyses (*, *P*=0.474/*P*=0.404, *P*=0.905/*P*=0.706, respectively) (Figure 4). The levels of TOS in the serum and kidney tissue were also examined. However, the serum and kidney samples from the ADR group demonstrated a substantial decrease in TAS levels as compared to the control and NAC groups (#, *P*=0.006; *P*=0.002, respectively) (Figure 4). Additionally, serum and kidney tissue TOS values significantly increased in the ADR group compared to the control and NAC groups (#, *P*=0.001; *P*=0.0325, respectively) (Figure 4) (Table 2).

ADR+NAC group had statistically substantially greater TAS levels in the serum and renal tissue than did the ADR group (#, *P*=0.010; *P*=0.010, respectively) (Figure 4). Furthermore, Table 2 shows that the TOS levels in the ADR+NAC group were considerably lower than those in the ADR group (Figure 4; #, *P*=0.009; *P*=0.004, respectively).

SPX levels in serum and kidney tissues were similar in the control group and NAC group (*P*=0.633; *P*=0.923, respectively) (Figure 4). 

In comparison to the control and NAC groups, the ADR group’s SPX levels significantly increased (Figure 4, *; *P*=0.023; *P*=0.009, respectively). The SPX levels in the ADR+NAC group, however, were lower than those in the ADR group (Figure 4; #, respectively; *P*=0.002; *P*=0.005) (Table 2).

## Discussion

ADR and other anthracyclines, classified as antitumor antibiotics, were discovered in the 1960s (30). These drugs exhibit high cytotoxicity against cancer cells but are limited in their usage due to significant side effects, notably cardiotoxicity, and nephrotoxicity. Nephrotoxicity is recognized as the primary adverse effect associated with ADR administration (31). ADR induces glomerular damage, tubular dilatation, atrophy, and histopathological changes, including renal fibrosis, directly affecting the kidney (32). Consistent with previous literature, our study observed histopathological alterations in kidney tissue following ADR exposure, such as edema, formation of hyaline casts, tubular dilatation, fibrosis, tubular vacuolization, and extravasation of erythrocytes.

The nephrotoxic effects of anthracycline antibiotics have been attributed to several mechanisms, as described in previous studies (33,34). Among these mechanisms, the induction of apoptosis and the generation of oxidative stress by ADR are considered the most significant and probable (35,36). The reduction of ADR can lead to the formation of various free radicals, which in turn cause DNA breaks, lipid peroxidation, and alcoholization of proteins and DNA (37). Insufficient levels of anti-oxidants are unable to provide adequate protection against ROS, resulting in mitochondrial damage and lipid peroxidation (38).

NAC, known for its potent anti-oxidant properties, exerts a protective effect on cells by inhibiting the formation of ROS and reducing apoptosis (39,40). Previous studies have reported that markers of kidney tissue damage, such as creatinine and BUN, increase with ADR exposure (2,41). Consistent with these findings, our study also observed a significant increase in BUN and creatinine levels following ADR exposure. Moreover, other studies have documented that ADR leads to renal oxidative damage by reducing anti-oxidant parameters and increasing oxidant parameters (42). Similar to this, our study found that following ADR administration, the levels of TAS and TOS increased in kidney tissue, indicating the existence of oxidative stress.

Previous studies have identified oxidative stress and apoptosis as the underlying pathological mechanisms of ADR-induced toxicity (43). In a separate study, diffuse apoptosis findings in tubular epithelial cells of the kidney tissue were reported in the ADR group using the TUNEL method (23). Consistent with the existing literature, our study also utilized the TUNEL method and confirmed that ADR administration resulted in apoptotic damage in kidney tissue. Another study investigating kidney damage reported that NAC treatment effectively increased TAS levels and concurrently decreased TOS levels in serum samples (44). This effect of NAC is believed to be primarily attributed to its anti-oxidant properties.

There is a scarcity of studies in the literature investigating the alterations in SPX immunoreactivity and serum/tissue SPX levels in different pathophysiological and histopathological conditions (45). Recent studies have shed light on the role of SPX in modulating the inflammatory process. For instance, it has been reported that SPX treatment can induce an inflammatory response in cases of chronic renal failure by regulating the levels of cytokines and chemokines (46). Similarly, another study observed that SPX administration improved renal oxidative stress and mitigated inflammation in obesity-induced renal dysfunction (19)

Several studies have highlighted the significant alterations in SPX levels in the pathogenesis of various diseases. Gu *et al*. suggested that SPX may play a crucial role in glucose and lipid metabolism in patients with type 2 diabetes (47). In a fructose-induced obesity model, SPX injection was found to significantly reduce the levels of pro-inflammatory cytokines (TNF-α, IL-1β, and IL-6) in the epididymal adipose tissue of mice (48). A study reported that intra-cerebroventricular administration of SPX exhibited a protective effect on kidney damage (49). Furthermore, in an adenine-induced chronic renal failure model, SPX administration demonstrated a potential protective effect against kidney damage and inflammatory processes (50).

Moreover, a recent study demonstrated that exposure to aluminum in rat kidney tissue induced histopathological changes, oxidative stress, apoptosis, and elevated SPX levels. The same study reported that NAC treatment mitigated the changes associated with aluminum exposure, including SPX levels, thereby potentially acting as a prophylactic measure against nephrotoxicity (22).

Consistent with the findings from previous studies, our study observed an increase in SPX immunoreactivity in kidney tissues as a result of ADR-induced nephrotoxicity. We speculate that this rise in SPX may help prevent kidney tissue from becoming damaged by oxidative stress and apoptosis caused by ADR. Consequently, while SPX levels were elevated in the ADR group, they exhibited a significant decrease in the ADR+NAC group, which corresponded to reduced markers of oxidative stress and apoptosis. This suggests that NAC treatment may have a therapeutic effect on ADR-induced nephrotoxicity by regulating SPX expression, which plays a crucial role in maintaining the balance between oxidative stress and apoptosis. Based on the observed changes in SPX levels in the injured and treatment groups, it can be inferred that SPX may contribute to the pathogenesis of ADR-induced kidney injury.

**Table 1:** Effect of ADR and/or NAC applications on histopathological evaluations, SPX Immunoreactivity and AI rate in rat kidney tissue

	Control Median (min-max)	NAC Median (min-max)	ADR Median (min-max)	ADR+NAC Median (min-max)	*p* ^*^
Edema	0.00(0.00-1.00)	^0.00(0.00-1.00)b^	^3.00(1.00-3.00)a^	^0.00(0.00-2.00)b^	= 0.001
Hyaline	0.00(0.00-0.00)	^0.00(0.00-0.00)b^	^3.00(2.00-3.00)a^	^0.00(0.00-1.00)b^	< 0.001
Tubular	^0.00(0.00-0.00)^	^0.00(0.00-0.00)b^	^2.00(1.00-3.00)a^	^0.00(0.00-1.00)b^	< 0.001
Fibrosis	^0.00(0.00-0.00)^	^0.00(0.00-0.00)b^	^2.00(1.00-3.00)a^	^0.00(0.00-1.00)b^	< 0.001
Tubular	^0.00(0.00-0.00)^	^0.00(0.00-0.00)b^	^ 3.00(2.00-3.00)a^	^0.00(0.00-1.00)b^	< 0.001
Erythrocyte	0.00(0.00-1.00)	^0.00(0.00-1.00)b^	^3.00(2.00-3.00)a^	^0.00(0.00-1.00)b^	< 0.001
Total histopathological evaluation score	0.00(0.00-2.00)	^0.00(0.00-2.00)b^	^12.00(10.00-16.00)a^	^0.00(0.00-5.00)b^	< 0.001
SPX	0.40(0.30-0.60)	^0.37(0.20-0.60)b^	^1.20(0.90-1.80)a^	^0.45(0.20-0.60)b^	= 0.003
AI	1.50(1.00-3.00)	^2.00(1.00-4.00)b^	^13.50(9.00-17.00)a^	^2.00(1.00-4.00)b^	= 0.003

The values were presented as median and min-max

a Compared to the control group, b Compared to the ADR group *P*<0.05), P* Kruskal Wallis

ADR: Adriamycin, NAC: N-acetylcysteine, AI: Apoptotic Index, SPX: Spexin.

Photomicrographs in Figure 1 show how the kidney tissues in various experimental groups look histologically. The control group (a) and NAC group (b) showed normal histological features without any noticeable abnormalities. In contrast, the ADR group exhibited various histopathological changes, including areas of erythrocyte extravasation (red arrow), tubular vacuolization (black arrow), edema (brown star), tubular dilatation (black star), and hyaline casts formation (notched black arrow) (c, d, e). However, it can be seen that the histopathological abnormalities seen in the ADR group were mitigated or reduced in the ADR+NAC group. (f). H&E, scale bar: 100 µm, x200 (a, b, c, d, e, f). ADR; adriamycin, NAC: N-Acetylcysteine, G: Glomerulus

Photomicrographs depict the histological appearance of kidney tissues in different experimental groups. The control group (a) and NAC group (b) exhibited normal histological features without any significant abnormalities. In the ADR group, areas of fibrosis (red asterisk) were observed, indicating tissue damage (c). However, it can be seen that the histopathological abnormalities seen in the ADR group were mitigated or reduced in the ADR+NAC group. (d). Masson Trichrome, scale bar: 100 µm, x200 (a, b, c, d,). ADR; Adriamycin, NAC: N-Acetylcysteine, G: Glomerulus

Figure 3 compares the immunoreactivity of SPX and the number of apoptotic cells discovered using the TUNEL technique in each group. The SPX immunoreactivity and TUNEL positivity levels in the control and NAC groups (a, b) were comparable, indicating no significant difference between them (*P*>0.05). However, the ADR group (c) exhibited a significant increase in SPX immunoreactivity and TUNEL positivity compared to the control and NAC groups, *P*<0.05). In the ADR+NAC group (d), there was a relative decrease in SPX immunoreactivity and TUNEL positivity compared to the ADR group *P*<0.05), suggesting that NAC treatment attenuated the ADR-induced increase in SPX immunoreactivity and reduced apoptotic cell death. It is worth mentioning that all slides were stained with methyl green for the TUNEL method. SPX immunohistochemical/TUNEL images, scale bar: 100 µm, ×200. G: Glomerulus, Arrow: SPX immunoreactivity/TUNEL positive cell, SPX: Spexin. Groups; ADR: Adriamycin, NAC: N-Acetylcysteine

The levels of creatinine, BUN, SPX, TOS, and TAS between the Control and NAC groups did not change significantly (*P*>0.05). Although creatinine, BUN, SPX, and TOS levels were significantly higher in the ADR group compared to the Control and NAC groups (*, *P*<0.05), TAS levels were significantly lower. On the other hand, there was a statistically significant decline in the levels of creatinine, BUN, SPX, and TOS in the ADR+NAC group compared to the ADR group, as well as a relative increase in TAS levels (#, *P*<0.05). *: *P*<0.05 compared to group Control; #: *P*<0.05 compared to group ADR

ADR: Adriamycin, BUN: Blood Urea Nitrogen, NAC: N Acetylcysteine, TOS: Total Oxidant Status, TAS: Total Anti-oxidant Status

**Table 2:** Effects of ADR and/or NAC applications on kidney function parameters, oxidative stress parameters and SPX levels in rat kidney tissue

	Control Median (min-max)	NAC Median (min-max)	ADR Median (min-max)	ADR+NAC Median (min-max)	*p* ^*^
Creatinin	0.66(0.56-0.73)	^0.67(0.62-0.74)b^	^0.92(0.81-0.98)a^	^0.73(0.61-0.82)b^	= 0.001
BUN	52.00(46.00-70.00)	^52.00(48.00-61.00)b^	^102.00(74.00-145.00)a^	^63.00(50.00-76.00)b^	= 0.001
TOS (pg/ml, * Serum*)	^51.15(45.62-56.38)^	^55.12(39.69-61.25)b^	^118.85(117.35-123.67)a^	^56.80(48.65-65.23)b^	= 0.004
TOS (pg/ml, * Tissue*)	^59.07(48.56-68.52)^	^55.00(38.96-62.10)b^	^120.32(118.45-122.45)a^	^56.07(42.56-68.78)b^	= 0.004
TAS (U/ml, * Serum*)	^5.35(4.03-7.30)^	^5.53(4.58-6.81)b^	^0.98(0.35-1.32)a^	^5.23(4.35-6.92)b^	= 0.005
TAS (U/ml, Tissue)	5.34(4.41-6.92)	^5.00(4.35-7.85)b^	^0.74(0.45-1.21)a^	^4.80(4.09-6.58)b^	= 0.004
SPX (pg/ml, * Serum*)	478.27(451.48-598.65)	^461.19(419.46-490.08)b^	^1745.18(1598.63-2154.02)a^	^449.39(365.23-574.83)b^	= 0.005
SPX (pg/ml, * Tissue*	447.70(387.44-474.07	^443.00(381.79-475.96)b^	^1548.62(1114.25-1993.12)a^	^443.47(391.21-494.79)b^	= 0.006

Values were presented as median and min-max

a Compared to the control group, b Compared to the ADR group (*P*<0.05), P* Kruskal Wallis

ADR: Adriamycin, BUN: Blood Urea Nitrogen, NAC: N Acetylcysteine, TOS: Total Oxidant Status, TAS: Total Anti-oxidant Status, SPX: Spexin

## Conclusion

Administration of ADR resulted in notable histopathological changes in the kidney tissue. Additionally, ADR exposure led to an increase in total oxidant levels, apoptotic cell counts, and SPX levels, while decreasing the total anti-oxidant level. However, treatment with NAC demonstrated significant improvement in all of these changes. Considering the observed alterations in SPX levels in the injury and treatment groups, it is plausible to suggest that SPX may play a role in the pathogenesis of ADR-induced kidney injury. Furthermore, we believe that further detailed studies investigating the pathophysiological mechanisms associated with kidney damage caused by ADR may help elucidate the treatment mechanisms related to SPX, to prevent or treat kidney damage more effectively in the future.

## Authors’ Contributions

İE O, T K, and T Y conceived the study; T K, T Y, and NK T designed the study; T Y, NK T, and A T performed data collection and processing; T K and T Y performed data analysis and interpretation; T Y reviewed the literature and drafted and wrote the manuscript; İE O, T K, T Y, and NK T critically reviewed the manuscript.

## Ethical Approval

All procedures involving animals in this study were conducted in compliance with the ethical standards of the institution or practice. The study received approval from the Fırat University Animal Experiments Ethics Committee, Turkey (granted on 09/04/2020, number 2020/05).

## Data Availability Statement

The data supporting the findings of this study belong to the Firat University, Faculty of Medicine, Department of Histology and Embryology. These data can be obtained from the corresponding author upon reasonable request.

## Funding

This study received support from Firat University Scientific Research Projects, Turkey (Project Number TF. 21.11).

## Conflicts of Interest

The authors declare no conflicts of interest.
